# Multiple waves of freshwater colonization of the three-spined stickleback in the Japanese Archipelago

**DOI:** 10.1186/s12862-020-01713-5

**Published:** 2020-11-03

**Authors:** Ryo Kakioka, Seiichi Mori, Tomoyuki Kokita, Takuya K. Hosoki, Atsushi J. Nagano, Asano Ishikawa, Manabu Kume, Atsushi Toyoda, Jun Kitano

**Affiliations:** 1 Ecological Genetics Laboratory, National Institute of Genetics, Mishima, Shizuoka 411-8540 Japan; 2 Present Address: Tropical Biosphere Research Center, University of the Ryukyus, Nishihara, Nakagami-gun, Okinawa, 903-0213 Japan; 3 Biological Laboratory, Gifu Kyoritsu University, Ogaki, Gifu 503-8550 Japan; 4 Department of Marine Bioscience, Fukui Prefectural University, Obama, Fukui 917-0003 Japan; 5 Department of Genetics, The Graduate University for Advanced Studies, Mishima, Shizuoka 411-8540 Japan; 6 Faculty of Agriculture, Ryukoku University, Otsu, Shiga 520-2194 Japan; 7 Field Science Education and Research Center, Kyoto University, Kyoto, 606-8502 Japan; 8 Comparative Genomics Laboratory, National Institute of Genetics, Mishima, Shizuoka 411-8540 Japan

**Keywords:** Restriction-site associated DNA sequencing, Convergent evolution, Glacial relic, Interglacial refugia, Non-native population, Hybridization, Speciation

## Abstract

**Background:**

The three-spined stickleback (*Gasterosteus aculeatus*) is a remarkable system to study the genetic mechanisms underlying parallel evolution during the transition from marine to freshwater habitats. Although the majority of previous studies on the parallel evolution of sticklebacks have mainly focused on postglacial freshwater populations in the Pacific Northwest of North America and northern Europe, we recently use Japanese stickleback populations for investigating shared and unique features of adaptation and speciation between geographically distant populations. However, we currently lack a comprehensive phylogeny of the Japanese three-spined sticklebacks, despite the fact that a good phylogeny is essential for any evolutionary and ecological studies. Here, we conducted a phylogenomic analysis of the three-spined stickleback in the Japanese Archipelago.

**Results:**

We found that freshwater colonization occurred in multiple waves, each of which may reflect different interglacial isolations. Some of the oldest freshwater populations from the central regions of the mainland of Japan (hariyo populations) were estimated to colonize freshwater approximately 170,000 years ago. The next wave of colonization likely occurred approximately 100,000 years ago. The inferred origins of several human-introduced populations showed that introduction occurred mainly from nearby habitats. We also found a new habitat of the three-spined stickleback sympatric with the Japan Sea stickleback (*Gasterosteus nipponicus*).

**Conclusions:**

These Japanese stickleback systems differ from those in the Pacific Northwest of North America and northern Europe in terms of divergence time and history. Stickleback populations in the Japanese Archipelago offer valuable opportunities to study diverse evolutionary processes in historical and contemporary timescales.

## Background

The presence of phylogenetically independent lineages adapting to similar environments offers great opportunities to investigate the roles of natural selection in phenotypic evolution [1]. Furthermore, such replicate systems enable us to investigate the extent to which causative alleles and genes are shared among independent lineages adapting to similar environments and what factors determine the probabilities of sharing the same alleles and genes [2–5]. Such knowledge will help to understand the repeatability and predictability of evolution [2–5]. Although several researchers distinguish between parallel and convergent evolution based on the underlying genetic mechanisms with the former caused by the same genetic mechanisms and the latter by different mechanisms, we call both parallel evolution in this study, because it is often difficult to draw a clear line between them [6].

Because transition from marine to freshwater habitats occurred in multiple lineages [7, 8], we can find replicate pairs of closely related marine and freshwater organisms. Marine and freshwater environments differ in many biotic and abiotic factors. Therefore, phylogenetically independent lineages that achieved the marine–freshwater transition would offer great opportunities to investigate the genetic basis for parallel/convergent evolution accompanying freshwater colonization and adaptation [7, 8].

Among the organisms that have undergone the marine–freshwater transition, the three-spined stickleback (*Gasterosteus aculeatus*) are a remarkable system to study the genetic mechanisms underlying this transition [9–11]. The three-spined stickleback is a cold-water fish widely distributed in coastal marine, brackish, and freshwater habitats of the Northern hemisphere [12, 13]. Ancestral marine ecotypes of the three-spined stickleback colonized freshwater habitats across its distribution. Many of these habitats emerged following deglaciation during the Quaternary Period. Freshwater populations from different geographic regions often show similar morphology and physiology. Thus, the three-spined stickleback is an excellent system to investigate the genetic mechanisms underlying parallel evolution [9, 10, 12–17].

Previous genetic studies on the parallel evolution of sticklebacks have mainly focused on postglacial freshwater populations in the Pacific Northwest of North America and in northern Europe [9–11]. The habitats in these regions were covered by ice sheets during the last glacial period and became uncovered within the last 12,000 years. Parallel evolution of several morphological and physiological traits in these postglacial populations has been caused by repeated fixation of identical-by-decent alleles [18–20]. Freshwater environments select freshwater-adaptive alleles that previously existed as standing variations in the founding marine populations [14, 18, 20–22], whose standing allelic variation may be maintained by gene flow from another freshwater population [9]. However, cases in which independent mutations of the same genes or different genes underlie parallel evolution have been described [10, 14, 15, 20, 21, 23].

Recent studies have demonstrated that geographically distant lineages, such as East Pacific and Atlantic lineages, use different sets of standing genetic variations for parallel evolution [21, 23]. These results indicate that analysis of geographically diverse regions can help to understand the wide distribution of freshwater-adaptive alleles in *G. aculeatus* across its distribution [21]. Such analyses can also clarify the alternative solutions when standing variations are not available [24, 25].

**Fig. 1:**
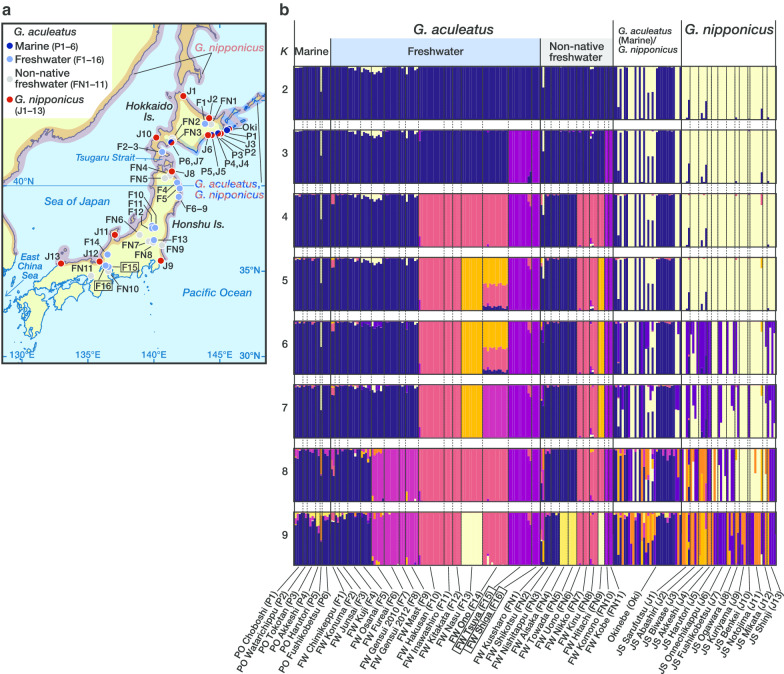
**a** Sampling sites in the Japanese Archipelago and distribution ranges of marine *Gasterosteus aculeatus* and *G. nipponicus*. See **b** for population codes. Distribution range of *G. nipponicus* is shaded in red. The distribution range of marine *G. aculeatus*, which overlaps with that of *G. nipponicus* in the Japanese Archipelago, is hatched in dark blue. Population codes enclosed in a box (F15 and F16) indicate the native habitats of the hariyo sticklebacks. The distribution ranges followed those of Higuchi [100], Higuchi et al. [33], Kitano and Mori [28] and Yoshigou [101]. The map was created with rnaturalearth ver. 0.1.0 (https://github.com/ropensci/rnaturalearth) and sf ver. 0.9-0 (https://r-spatial.github.io/sf/). **b** Bar plots showing the results of the population structure analyses of Japanese samples based on 2735 SNPs with ADMIXTURE (*K* = 2–9). Individuals are represented as vertical bars proportional to the genotypes belonging to each of the genetic clusters. Population codes in brackets follow population names. Population names enclosed in a box (FW Tsuya [F15] and FW Shiga [F16]) indicate the hariyo sticklebacks

Japanese three-spined stickleback populations in the western Pacific basin offer several unique opportunities to investigate the genetic basis of parallel evolution (Fig. 1a). First, the Japanese Archipelago is geographically distant from North America and Europe, suggesting that the Japanese populations may share a relatively small number of genetic variants with North American and European populations. Previous studies have shown that reduction in the armor plate in freshwater populations in North America and Europe is caused by repeated fixation of the same *ectodysplasin* (*Eda*) allele, whereas armor plate reduction in Japanese freshwater populations is caused by independent mutations at *Eda* [9, 14, 24, 26].

Second, there are freshwater populations with different ages of colonization. The Japanese Archipelago was not covered by ice sheets in the Quaternary glaciation, suggesting that several freshwater habitats were accessible by sticklebacks well before 12,000 years ago. A previous mitochondrial DNA phylogenetic analysis estimated the divergence time of freshwater populations in Gifu and Shiga, central Honshu Island, termed “hariyo stickleback” in Japan [27, 28], from the rest of *G. aculeatus* as 0.37–0.43 million years before present (Ma BP) based on a molecular clock. Additionally, there are several young freshwater populations, e.g. those inhabiting lakes and ponds that were formed within 2000–3000 years in eastern Hokkaido [29, 30]. These freshwater populations are not genetically differentiated from marine *G. aculeatus* at allozyme or microsatellite loci [29, 30]. Several human-introduced populations also offer opportunities to investigate the genetic basis of rapid adaptation [31, 32]. Freshwater populations with such a diverse array of colonization ages provide opportunities to investigate how freshwater adaptation progresses over time.

Finally, the distribution range of *G. aculeatus* overlaps with that of its sister species *G. nipponicus* in northern Japan [33, 34]. Previous studies have shown that all freshwater populations examined thus far belong to *G. aculeatus* rather than *G. nipponicus* [15, 35]. *G. aculeatus* has higher copy numbers of the metabolic gene *Fads2* and can survive better on freshwater-derived diets than *G. nipponicus* [15]*.* Because there is past and ongoing hybridization between these two species [32, 36–38], it is important to determine the extent of introgression of freshwater-adaptive alleles between these two species to understand the genetic factors constraining the freshwater colonization of *G. nipponicus*.

As a first step towards a comprehensive understanding of the genetic basis of parallel evolution in the Japanese freshwater populations of *Gasterosteus*, we investigated their origins using phylogenomic approaches. The majority of previous phylogenetic studies on Japanese sticklebacks have used allozyme, microsatellite, and mitochondrial DNA. Mitochondrial DNA has been shown to introgress from *G. nipponicus* to *G. aculeatus*, suggesting that phylogeny based on mitochondrial DNA does not reflect the population history [37, 39, 40]. Previous phylogenetic analyses using allozyme and microsatellite were based on a small number of markers. More precise phylogenetic analysis with a large number of genome-wide single nucleotide polymorphisms (SNPs) are necessary. We have conducted phylogenetic analyses using whole genome sequences [41] and Restriction-site associated DNA (RAD) markers [15]. However, we have identified several new habitats of freshwater populations and new possible hybrid zones between *G. acuelatus* and *G. nipponicus* since then. Additionally, previous studies did not investigate the divergence time or phylogenetic relationships of the Japanese populations with North American and European populations. To solve these unanswered questions, we conducted a phylogenomic analysis of Japanese stickleback populations using RAD sequencing.

## Results

### Population structure

Two clusters revealed by ADMIXTURE analysis of all samples from the Japanese Archipelago at *K* = 2 reflected interspecies differentiation between *G. aculeatus* and *G. nipponicus* (Fig. 1b). All freshwater populations, including the artificially introduced non-native ones, were assigned to the *G. aculeatus* cluster. Although *G. aculeatus* and *G. nipponicus* were overall genetically differentiated, hybrids were also found at several localities. If we judge fish with *Q* values (admixture proportion of the ADMIXTURE analysis) < 0.875 for either species as hybrids, such hybrids were mostly found in marine populations, although freshwater populations in Otsuchi (FW Fureai [population code: F6], FW Mast [F9]) and a non-native population, FW Kussharo (FN1), also contained hybrids.

Increasing *K* identified more freshwater clusters and revealed genetic distinctiveness among freshwater populations. At *K* = 3, the hariyo stickleback, FW Tsuya (Gifu; F15) and FW Shiga (F16), separated from other *G. aculeatus* populations. Two introduced populations (FW Komono [FN10], FW Kobe [FN11]) were assigned to this cluster. At *K* = 4, several freshwater populations, Aizu populations (FW Hakusan [F10], FW Inawashiro [F11], FW Kitakata [F12]), FW Nasu (F13), and FW Ono (F14), separated as a cluster from the rest of the native *G. aculeatus* populations. At *K* = 5, populations from Aizu (FW Hakusan [F10], FW Inawashiro [F11], FW Kitakata [F12]) further separated from this cluster, to which non-native populations of FW Uono (FN6), FW Nikko (FN7), and FW Kinu (FN8) also belonged. Individuals of FW Nasu (F13) formed a distinct cluster together with the non-native FW Hitachi (FN9) population at *K* = 7 and 9. The cross-validation error was the lowest at *K* = 8 (Additional file 1, Fig. S1). At *K* ≥ 6, *G. nipponicus* contained distinct clusters, but the clustering was incongruent among different *K*.

Results of the ADMIXTURE analyses were supported by principal component analyses (PCA) (Additional file 2, Fig. S2). PC1 separated *G. aculeatus* and *G. nipponicus*. The hybrids identified in the ADMIXTURE analysis were placed between *G. aculeatus* and *G. nipponicus*. PC2 separated the hariyo stickleback, FW Tsuya (Gifu; F15) and FW Shiga (F16), from the other populations. The freshwater population of FW Nasu (F13) was distinct along the PC3 axis. PC4 splits the Aizu populations (FW Hakusan [F10], FW Inawashiro [F11], FW Kitakata [F12]), FW Ono (F14), and others. Grouping of non-native populations to native populations was concordant with the assignment of the ADMIXTURE clustering.

### Phylogeny

**Fig. 2:**
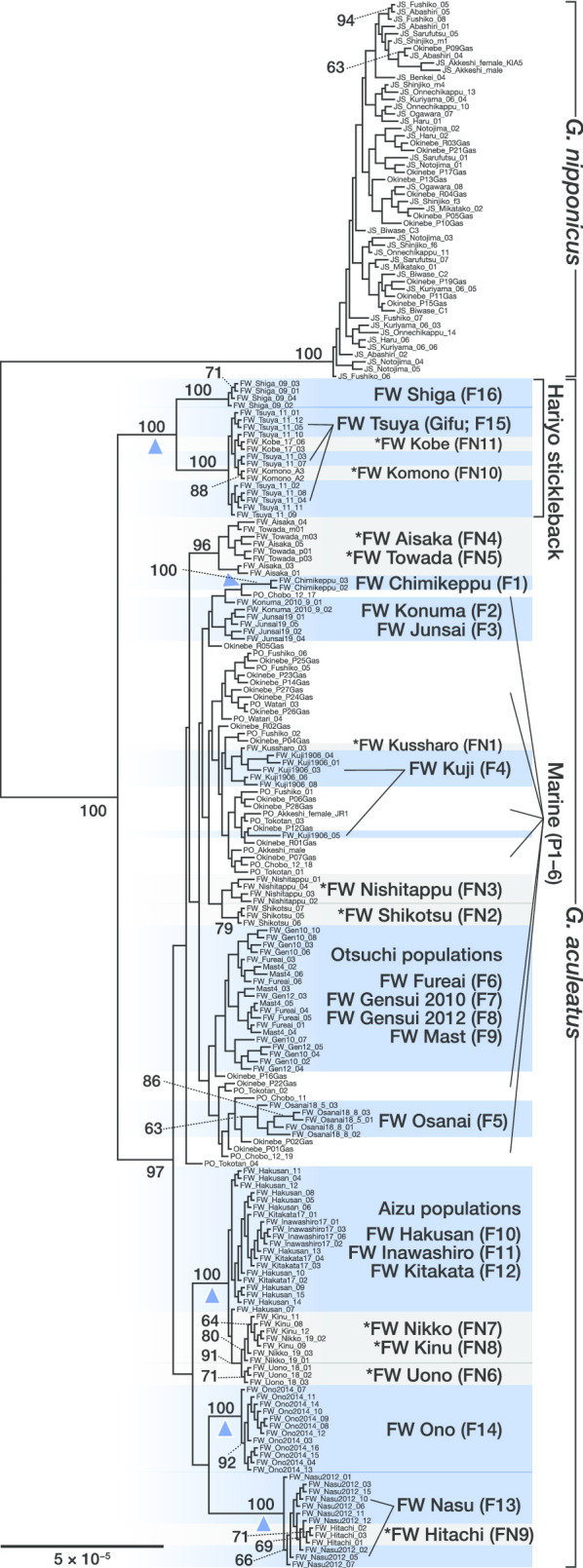
Maximum likelihood phylogenetic tree of non-hybrid individuals from Japan based on 1919 concatenated SNPs. Bootstrap values (> 60%) are shown. Individuals from native freshwater populations of *Gasterosteus aculeatus* are highlighted in blue and those from non-native populations of *G. aculeatus* are highlighted in grey. Non-native population names are marked with asterisks. Blue arrowheads indicate monophyletic freshwater populations and a group of freshwater populations in vicinity

Maximum likelihood (ML) phylogeny using concatenated SNPs of the Japanese samples clearly distinguished *G. nipponicus* and *G. aculeatus* (Fig. 2). The hariyo stickleback were monophyletic and first branched off from the rest of *G. aculeatus*. Another monophyletic clade composed of populations from the Aizu Basin (FW Hakusan [F10], FW Inawashiro [F11], FW Kitakata [F12]), FW Nasu (F13), and FW Ono (F14) split from the rest of *G. aculeatus*. Other freshwater populations were not monophyletic and nested in marine populations. The placement of non-native populations was congruent with the results of the population structure analyses. Non-native populations from adjacent sites often clustered together. These comprised FW Shikotsu (FN2) and FW Nishitappu (FN3); FW Aisaka (FN4) and FW Towada (FN5); and FW Uono (FN6), FW Nikko (FN7), and FW Kinu (FN8).

**Fig. 3:**
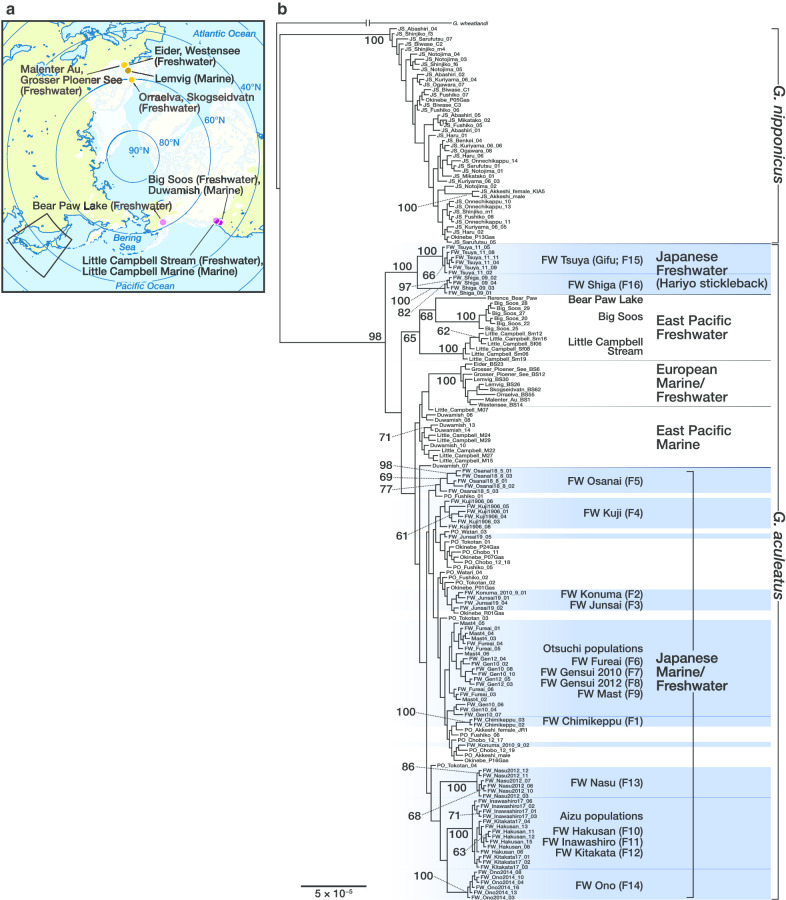
**a** Sampling sites of *Gasterosteus aculeatus* in the eastern Pacific basin and northern Europe. The extent of Fig. 1a is bounded with black lines. The configuration of ice sheets at the last glacial maximum [102] is shown with white shading. The map was created with rnaturalearth ver. 0.1.0 (https://github.com/ropensci/rnaturalearth) and sf ver. 0.9-0 (https://r-spatial.github.io/sf/). **b** Maximum likelihood phylogenetic tree of native non-hybrid individuals from western and eastern basins of the Pacific and northern Europe based on 3717 concatenated SNPs. Individuals from Japanese freshwater populations of *G. aculeatus* are highlighted in blue. Bootstrap values (> 60%) are shown

ML phylogenetic analysis including the samples from the western and eastern basins of the Pacific and northern Europe (Fig. 3) also supported the monophyly of the hariyo stickleback, which split from the rest of *G. aculeatus* earlier than any other freshwater populations examined (Fig. 3b). Next, populations from the East Pacific and Europe branched off. All of the Japanese *G. aculeatus* populations other than the hariyo stickleback were monophyletic, although the bootstrap support was low (bootstrap value < 60%).

**Fig. 4:**
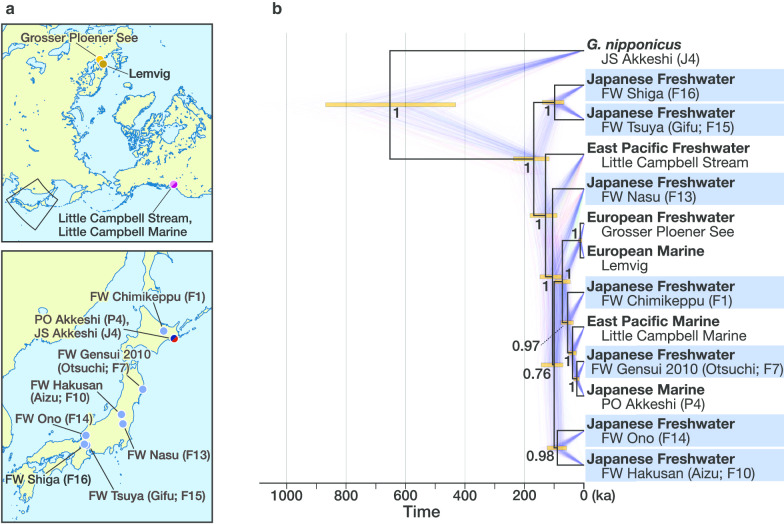
**a** Sampling sites of *Gasterosteus aculeatus* used for a time-calibrated species tree analysis. **b** A time-calibrated species tree of representative populations of *Gasterosteus aculeatus* and *G. nipponicus* inferred with SNAPP based on 2022 SNPs with the root calibration at 680 ka. The trees recorded in a run are overlaid by the maximum clade credibility tree. Posterior probabilities of each node are shown. Each bar plot indicates the 95% highest posterior density interval of the node height. Individuals from Japanese freshwater populations of *G. aculeatus* are highlighted in blue. The results of other runs are provided in Additional file 3, Fig. S3

The topology of the species tree obtained by the SNAPP analyses was identical among all the runs and were generally congruent with the ML trees (Fig. 4, Additional file 3, Fig. S3). Most of the nodes were strongly supported with posterior probabilities of > 0.93, except for one (see the node with 0.76 in Fig. 4). The divergence times of each node agreed well among the runs with the same prior on divergence time and were scalable to the root divergence time with different priors. Assuming a root divergence of 680 thousand years (ka) before present (BP), which was estimated by a demographic analysis with an approximate Bayesian computation approach [37], the mean divergence time between *G. aculeatus* and *G. nipponicus* was estimated to be 644–653 ka (95% highest posterior density intervals [95HDI] = 395–868 ka) (for the results of other runs, see Additional file 3, Fig. S3B and C). For the results assuming divergence at 1.38 million years (Ma) BP, see Additional file 3, Fig. S3D–F. Hariyo stickleback diverged from the rest of *G. aculeatus* at 167–169 ka (95HDI = 114–237 ka) BP. Two lineages within the hariyo stickleback, FW Tsuya (Gifu; F15) and FW Shiga (F16), diverged at 97–99 ka (95HDI = 62–140 ka) BP. The divergence time of a Japanese freshwater population FW Nasu (F13) from the rest was 104–106 ka (95HDI = 65–148 ka), while the freshwater lineage leading to FW Hakusan (Aizu; F10) and FW Ono (F14) diverged at 96–100 ka (95HDI = 59–144 ka) BP. FW Chimikeppu (F1) diverged at 55 ka (95HDI = 35–79 ka) BP, while a younger Japanese freshwater population from Otsuchi (FW Gensui 2010 [F7]) diverged at 24 ka (95HDI = 14–34 ka) BP.

As for the North American and European populations, the divergence of an East Pacific freshwater population (Little Campbell Stream) from the rest occurred at 128–129 ka (95HDI = 76–182 ka) BP. European and Pacific populations excluding this old Pacific freshwater lineage diverged 72–73 ka (95HDI = 45–99 ka) BP. The East Pacific marine population from the estuary of Little Campbell River diverged from the Japanese Pacific Ocean marine population 38–39 ka (95HDI = 22–53 ka) BP. North European freshwater (Grosser Ploener See) and marine (Lemvig) populations diverged 13 ka (95HDI = 7–19 ka) BP.

## Discussion

### Multiple waves of freshwater colonization in the Japanese three-spined sticklebacks

Our results based on genome-wide SNPs with new additional populations support previous findings that all freshwater populations in the Japanese Archipelago are within the *G. aculeatus* clade [15, 34, 35]. Furthermore, our present phylogenetic analysis showed that the Japanese freshwater populations are not monophyletic, suggesting that freshwater colonization has occurred in multiple waves.

Freshwater populations called hariyo sticklebacks are the oldest extant freshwater lineages of the species reported thus far. Phylogenetic analyses revealed the monophyly of the hariyo sticklebacks. Bayesian species tree analysis showed that the divergence of the hariyo sticklebacks from the rest of *G. aculeatus* was approximately 167–169 ka BP, which largely predates the last glacial period and is the oldest extant freshwater lineage ever reported. The eastern Pacific basin harbors old freshwater lineages [42, 43] and the present data confirmed that the divergence time of a stream population from the eastern Pacific (Little Campbell River) predates the end of the last glacial period. A previous study using a SNAPP species tree analysis based on the same calibration point with similar priors [42] estimated the divergence time of another freshwater population from the East Pacific basin (Beaver Lake on Vancouver Island) as 119 ka BP, which is close to our estimate of the divergence time of the eastern Pacific freshwater population. Nonetheless, the divergence of the hariyo stickleback lineage preceded that of the eastern Pacific stream populations. To date, no previous phylogenetic analysis at the global scale using genome-wide SNP data (e.g., [18, 43]) have included the hariyo lineage.

The freshwater lineages of Nasu (FW Nasu [F13]), Aizu (FW Hakusan [F10], FW Inawashiro [F11], FW Kitakata [F12]), and Ono (FW Ono [F14]) were estimated to have diverged at approximately 100 ka BP. This is still before the latest Pacific–Atlantic split, which has been suggested to have occurred when the Bering Strait closed somewhere between 34 and 75 ka BP during the last glacial period [44]. Other freshwater lineages in northern Japan have diverged more recently. FW Gensui 2010 (F7) from Otsuchi was estimated to have diverged at approximately 24 ka BP, which is close to the time of postglacial freshwater colonization in northern Europe [42].

Interglacial isolations can explain some of these multiple waves of freshwater colonization in the Japanese three-spined stickleback. Sticklebacks favor a cooler climate [12, 45], so they would shift the distribution southward during glacial periods and northward during interglacial periods [41]. Freshwater populations in central Honshu Island are presently restricted to springs and spring-fed streams in which water temperature is maintained below 20 °C, allowing the fish to avoid heat in summer [28, 45]. Habitats of the hariyo stickleback and the Nasu population are on the Pacific slope, out of the current distribution range of marine *G. aculeatus*. The waters inhabited by the Aizu and Ono populations are drained by the rivers that flow into the Sea of Japan, where *G. aculeatus* is absent at present (Fig. 1a). In addition to global cooling, southward extension of the cold ocean current in the Pacific Ocean 12.8–21 ka BP [46], shut-off of the warm Tsushima Current from the East China Sea into the Sea of Japan during glacial periods [47], and the intrusion of the cold Oyashio Current into the Sea of Japan through the Tsugaru Strait 4.8–17.5 ka BP [48] could have shifted the range of marine *G. aculeatus* southward during the last and preceding glacial periods. The collective data support the hypothesis that these freshwater lineages are glacial relicts originating from ancient marine *G. aculeatus* that once shifted its distribution southward during the glacial periods. California in the eastern Pacific basin also houses isolated freshwater populations, which may have colonized before the last glacial period [49, 50]. Some freshwater populations from southern Europe may also be glacial relics [42, 43, 51–55]. Therefore, freshwater colonization and subsequent isolation in the glacial–interglacial cycles likely have come in multiple waves at multiple geographical regions across the distribution range of the three-spined stickleback.

Although the hariyo sticklebacks may be the oldest extant freshwater lineage, fossils of *Gasterosteus* from both the eastern and western basins of the Pacific date back to 10 Ma BP [56–58]. This suggests that *Gasterosteus* flourished around the Pacific, including fresh waters, since at least 10 Ma BP [57]. These fossils largely predate the divergence of the hariyo lineage of *G. aculeatus* and even the split of *G. aculeatus* and *G. nipponicus* [37]. Although these ancient freshwater *Gasterosteus* are not direct ancestors of the extant freshwater populations of *G. aculeatus*, they may have served as sources of standing variation of freshwater-adaptive alleles that have facilitated freshwater adaptation in extant *G. aculeatus* [9, 18]. The ancient age (average of 6.4 Ma) of several freshwater-adaptive alleles segregating in extant *G. aculeatus* [59] is consistent with this idea. Analysis of standing genetic variation of these freshwater-adaptive alleles in the Japanese marine and freshwater populations will provide insights into how widely freshwater-adaptive alleles are shared among global populations in *Gasterosteus* and what genetic mechanisms have enabled freshwater adaptation in parallel.

### Non-native populations

Recent human activities have moved sticklebacks from original habitats to non-native habitats. Our genetic analysis showed that the introduced populations were derived from nearby habitats. For example, non-native populations from FW Komono (FN10) and FW Kobe (FN11) clustered with the nearby FW Tsuya (F15). All of these are located in southwestern Honshu Island. Non-native populations from northern Japan in Hokkaido (FW Kussharo [FN1], FW Shikotsu [FN2], and FW Nishitappu [FN3]) and northern Honshu Islands (FW Aisaka [FN4] and FW Towada [FN5]) were genetically similar to *G. aculeatus* distributed in northern Japan. Non-native populations from central Honshu (FW Uono [FN6], FW Nikko [FN7], FW Kinu [FN8], and FW Hitachi [FN9]) were derived from either Aizu or Nasu populations. Although non-native populations can provide opportunities to study the process of adaptation to novel habitats on a contemporary timescale [31, 32], their spread may lead to hybridization with, or extinction, of native populations [28]. Native freshwater populations are invaluable genetic resources to study the genetic basis of adaptive phenotypic diversification generated during the last 200,000 years in the Japanese Archipelago. Thus, it is important to conserve them. Particular caution is needed to prevent translocation of sticklebacks between water systems, which can lead to genetic contamination or even population extinction due to hybridization [60].

### A new sympatric habitat

In addition to previously reported sympatric habitats [30, 34, 36–38, 61, 62], we identified a new sympatric habitat of *G. aculeatus* and *G. nipponicus* at the eastern end of Hokkaido (Okinebe [Oki]). Based on the *Q* values of the ADMIXTURE analysis, among 32 fish analyzed, two individuals were F_1_ hybrids and one was a backcross to *G. nipponicus*. Okinebe Pond is relatively small (approximately 30,000 m^2^) and is connected to the Pacific Ocean by a short stream approximately 200 m in length. The frequency of hybrids in this pond is relatively high compared to previously investigated sympatric habitats. Previous genomic studies have shown that sympatric habitats can differ in the magnitude of reproductive isolation and hybridization [37, 38]. This new sympatric habitat would provide an additional study system to investigate the genetic and ecological mechanisms underlying reproductive isolation between these two species.

## Conclusions

Stickleback populations in the Japanese Archipelago offer valuable opportunities to study a wide spectrum of evolutionary processes in historical and contemporary timescales. First, Japanese freshwater populations provide phylogenetically independent and geographically distant replicates of stickleback freshwater populations. Using these systems, we can test the extent to which causative alleles and genes are shared among independent lineages adapting to similar environments and what factors determine the probabilities of sharing the same alleles and genes [2–5]. Second, several newly identified non-native populations will provide us opportunities to investigate the genetic and ecological mechanisms underlying rapid evolution [63]. Finally, replicates of sympatric habitats of *G. aculeatus* and *G. nipponicus* enable us to test whether the same genomic loci are resistant to introgression or likely to introgress between closely related species [38]. By characterizing these loci, we can obtain insights into the genomic patterns of divergence and introgression during speciation with gene flow [64, 65]. In conclusion, Japanese stickleback populations provide a valuable system to study the genetic basis of adaptation and speciation.

## Methods

### Sample collection

All sticklebacks were collected with seine nets and minnow traps as described previously [20, 30–32, 37, 66, 67] (Fig. 1a). After euthanasia with an overdose of MS-222 (0.5 g/L), the pectoral fins were dissected out and preserved in 99% ethanol until use. Additional file 4, Table S1 and Additional file 5, Table S2 provide details of the samples. Morphologically identified species [33] collected at the same locality were denoted as different populations, with the exception of Okinebe (Oki), where *G. aculeatus*, *G. nipponicus*, and possibly their hybrids are supposed to be included.

### Laboratory experiments and sequencing

Genomic DNA was isolated using a DNeasy Blood & Tissue Kit (QIAGEN, Valencia, CA, USA). Double digest RAD sequencing (ddRAD-seq) was performed as described previously [68]. Briefly, 10 ng of genomic DNA was digested with EcoRI and BglII, followed by adapter ligation and amplification with uniquely barcoded primers. The libraries were run on HiSeq 2000 or 2500 using the 50 bp single-end or a 100 bp paired-end mode at Macrogen (Kyoto, Japan) or the Advanced Genomics Center of the National Institute of Genetics (Shizuoka, Japan). The sequence data are available from DDBJ/EMBL-EBI/NCBI Sequence Read Archive (DRA010673). Some of the ddRAD-seq data has been published previously [15] (see Additional file 5, Table S2).

Additionally, we used publicly available whole genome sequence (WGS) data (Additional file 5, Table S2). For *G. aculeatus* collected from PO Akkeshi (P4), *G. nipponicus* from JS Akkeshi (J4), and *Gasterosteus wheatlandi*, we used the previously reported whole genome sequences [69]. Sequence data of *G. aculeatus* from FW Aisaka (FN4) and FW Towada (FN5) were derived from a previous study [32]. For *G. aculeatus* from northern Europe, the sequences of two randomly selected samples from the marine population reported previously [70], and those of one or two randomly selected samples from each freshwater population reported previously [71] were obtained.

### Sequence data processing

The flow of bioinformatic analyses is summarized in Additional file 6, Fig. S4. Trimming of ddRAD-seq reads was performed to remove adapter sequences and failed reads using Trimmomatic v0.39 [72] with the following parameters: “ILLUMINACLIP:TruSeq3-PE-2.fa:2:30:10:2 CROP:50 LEADING:3 TRAILING:3 MINLEN:50”. The trimmed reads were mapped to the BROADS S1 stickleback reference genome sequence (soft-masked, Ensembl 99) using NextGenMap v0.5.5 [73]. Variants were called with FreeBayes v1.3.2 [74], skipping sites with the average coverage per sample exceeding 500 and with the options: “-report-monomorphic-use-mapping-quality-use-best-n-alleles 8”. Sites of a sample with a coverage of less than five were discarded with BCFtools v1.9 [75].

We further selected RAD loci with the following criteria using BCFtools and bedtools v2.17.1 [76]. First, the sites genotyped in less than 25% of the samples and located on the mitochondrion were excluded. Next, we searched for the regions consecutively genotyped for at least 40 bp, allowing gaps not longer than 10 bp. The records within the identified RAD regions were extracted and variant representations were normalized with vt v0.5772 [77].

WGS reads were trimmed using Trimmomatic with the following settings: “ILLUMINACLIP:TruSeq3-PE-2.fa:2:30:10:2:true LEADING:3 TRAILING:3 MINLEN:30”. Overlapped paired-end reads were merged with PEAR 0.9.10 [78] and read pairing was confirmed with fastq-pair v1.0 [79]. Mate-pair reads from Feulner et al. [70] were reversed and complemented using SeqKit 0.10.0 [80]. The reads were mapped to the BROADS S1 stickleback reference sequence using NextGenMap v0.5.5. The maximum insert size for the alignments of the mate-pair reads was set to 6000. Duplicate reads were marked with Picard Tools v2.21.8 [81]. Variants within the selected contiguous RAD loci (see above) were called with FreeBayes using the same settings as that of ddRAD-seq. Sites of a sample with a coverage of less than five were discarded with BCFtool, and normalization of variants was conducted with vt.

The pre-processed variant calls from ddRAD-seq and WGS were merged using BCFtools. Block substitutions were decomposed into their constituent SNPs using vt. Indels, invariant sites, and sites on the sex chromosomes of *G. aculeatus* and *G. nipponicus* (Chromosomes IX and XIX) or those in masked regions or on ambiguous nucleotides in the reference sequence were discarded. Samples with excessively missing genotypes (> 80%) were excluded with BCFtools. This process resulted in a dataset of 97,145 SNPs genotyped in a total of 310 samples.

### Population structure analyses

In order to investigate genetic differentiation and potential introgression among the stickleback populations in the Japanese Archipelago, we first used a model-based likelihood clustering algorithm implemented in ADMIXTURE v1.3.0 [82]. We selected biallelic SNPs that were genotyped in all populations with only one missing population allowed, and that were missing in less than 30% of the overall samples with VCFtools v 0.1.17 [83]. If an allele at a SNP site was found in only one sample, the SNP site was excluded regardless of whether it was identified as “singleton” or “doubleton” with VCFtools. The SNPs were subsampled with VCFtools to maintain a minimum distance of 1 kb to reduce the effect of linkage between SNPs. The input file for ADMIXTURE including 2735 SNPs was created using PLINK v1.90 [84]. ADMIXTURE was run by varying the number of evolutionary clusters *K* from one to nine. The results were summarized and visualized using CLUMPAK [85] on the web (https://clumpak.tau.ac.il/index.html).

We also conducted principal component analyses (PCA), using the adegenet v2.1.1 package [86, 87] of R [88]. The dataset for the ADMIXTURE analysis was further filtered, keeping SNPs with minor allele frequency ≥ 0.03 and individuals with missing genotypes < 20%. This resulted in a dataset of 813 SNPs.

### Phylogenetic analyses

Maximum likelihood (ML) phylogenetic trees were constructed with RAxML-NG v0.9.0 [89] based on two datasets of concatenated SNPs. The first includes 1,919 SNPs of all the samples from Japan excluding putative recent hybrids between *G. aculeatus* and *G. nipponicus*, which would violate basic assumptions of phylogenetic reconstruction methods and bias tree topology and branch lengths. Hybrid individuals were identified using the ADMIXTURE analysis described in the section of Population structure analyses based on *Q* values assuming *K* = 2. When both a *Q* value for the *G. aculeatus* cluster and that for the *G nipponicus* cluster at *K* = 2 were < 0.875, that individual was classified as a hybrid. The identified hybrids were concordant with those detected by PCA (Additional file 2, Fig. S2, left panel).

The second tree consists of 3717 SNPs of non-hybrid individuals from native populations of western and eastern basins of the Pacific and Europe, which were subsampled to include at most six individuals per population, two of which had the least missing genotypes and the rest of which were randomly selected. Hybrids in the East Pacific populations were identified and excluded in a manner similar to that of Japanese samples using ADMIXTURE. We selected biallelic SNPs that were genotyped in all four populations using BCFtools. The SNPs were subsampled with VCFtools to maintain a minimum distance of 1 kb. ADMIXTURE was run by varying the number of evolutionary clusters *K* from one through four. Identification of hybrid individuals was conducted based on *Q* values from the ADMIXTURE analysis assuming *K* = 3. If the *Q* values of an individual for any cluster did not exceed 0.875, it was classified as a hybrid. As a result, three putative hybrids from Duwamish and Big Soos were removed (Additional file 7, Fig. S5). The reference sequence obtained from a freshwater stickleback collected at Bear Paw Lake, Alaska [18] was added as a sample in the second dataset. Each dataset included the SNPs genotyped in all but one population and > 70% of the overall samples, keeping the minimum distance between SNPs at 1 kb. We used the general time-reversible model of nucleotide substitution with gamma-distributed rate heterogeneity and ascertainment bias correction [90] using the conditional likelihood method [91]. We conducted bootstrap analyses with 200 replicates and searched for the best scoring trees in each of the two runs. The tree was visualized with FigTree v1.4.4 [92].

Phylogeny and divergence time among stickleback populations was estimated with the multispecies coalescent model using the Bayesian framework of SNAPP v1.5.0 [93] implemented in Beast v2.6.2 [94]. To reduce the computational time, we selected two non-hybrid individuals with the least missing genotypes from 13 representative populations covering the distribution range and distinct lineages of the stickleback. They consisted of *G. nipponicus*, marine populations of *G. aculeatus* from the western and eastern basins of the Pacific and Europe, freshwater populations from each of the three regions, including those comprising highly supported clades in the Japanese Archipelago that were revealed by the ML tree analysis. We removed SNPs with missing genotypes, and subsampled SNPs to maintain a minimal distance of 1 kb. This resulted in a dataset of 2022 biallelic SNPs.

Root divergence was used as the calibration point. We adopted two previously published estimates as the time of divergence between *G. aculeatus* and *G. nipponicus*. The first is 680 thousand years (ka) BP following our previous study [37], estimated by a demographic analysis with an Approximate Bayesian Computation approach. The second was the 1.38 Ma BP [43] based on a Bayesian estimation of phylogeny and divergence time with concatenated RAD sequences. Although the potential overestimation of the latter due to incomplete lineage sorting is pointed out [42], we included it to account for uncertainty in the estimation of the divergence time, since it is close to another estimate of 1.22 Ma BP based on an ML-based demographic analysis [37], and within the 95% confidence interval of the former divergence time estimate (0.18–4.1 Ma).

Prior for the divergence time was specified to follow a log-normal distribution with means in real space to the respective divergence times (i.e., 0.68 and 1.38 Ma), and with a standard deviation of 0.18 so that 95% intervals of the two priors do not overlap. We fixed a population parameter theta, which is proportional to the product of effective population size and mutation rate per site, to be equal across lineages with a uniform prior, following Stange et al. [95]. It should be noted that fixed and equal population sizes among all populations could flaw divergence time estimates obtained in the coalescent analysis. Monophyly of *G. aculeatus* (i.e., all the populations except *G. nipponicus*) and that of two European populations were set as constraints. We used a script by Matschiner [96] to prepare input files for SNAPP. Three independent runs were performed for each calibration scheme with a chain length of 1.54–2.22 × 10^6^ generations starting from different initial trees. Trees were sampled every 5000 steps and checked for convergence to the stationary distribution and a sufficient effective sample size (ESS > 200) using Tracer v1.7.1 [97]. The first 10% of the trees were discarded as burn-in and the remaining trees were visualized using DensiTree v2.2.7 [98]. Maximum clade credibility consensus trees of each run after burn-in were summarized with TreeAnnotator v2.6.2 [99] and visualized with FigTree [92].

## Supplementary information

**Additional file 1: Fig. S1.** Cross-validation errors for each *K* from the ADMIXTURE analyses for Japanese populations.

**Additional file 2: Fig. S2.** Scatter plots of principal components of genetic differentiation in the Japanese populations based on 813 SNPs. The contributions of each principal component are shown in the parentheses.

**Additional file 3: Fig. S3.** Time-calibrated species trees of representative populations of *Gasterosteus aculeatus* and *G. nipponicus* inferred with SNAPP based on 2022 SNPs. Results of three independent runs are shown. The trees recorded in a run are overlaid by the maximum clade credibility tree. Posterior probabilities of each node are shown. Each bar indicates the 95% highest posterior density interval of the node height. (A, B, and C) Calibrated with root divergence at 680 ka BP. (D, E, and F) Calibrated with root divergence at 1.38 Ma BP. Individuals from Japanese freshwater populations of *G. aculeatus* are highlighted in blue.

**Additional file 4: Table S1.** Information of collection sites.

**Additional file 5: Table S2.** Sample information

**Additional file 6: Fig. S4.** Summary of the flow of bioinformatic analyses.

**Additional file 7: Fig. S5.** (A) Bar plots showing the results of the population structure analyses of East Pacific samples based on 3790 SNPs with ADMIXTURE (*K* = 2–4). Individuals are represented as vertical bars with different colours being proportional to the genotypes belonging to each genetic cluster. (B) Cross-validation errors for each *K* from the ADMIXTURE analyses of the East Pacific populations.

## Data Availability

The sequence data generated during the current study are available in DDBJ (DRA010673).

## Abbreviations

ML: Maximum likelihood
*Eda*: *Ectodysplasin*
ddRAD: Double digest restriction-site associated DNA
SNP: Single nucleotide polymorphism
PCA: Principal component analysis
ka: Thousand years
Ma: Million years
BP: Before present
HDI: Highest posterior density intervals
