# Supplementary figures and images for: Multiple waves of freshwater colonization of the three-spined stickleback in the Japanese Archipelago
Source: BMC Evol Biol. 2020 Nov 3;20:143. doi: 10.1186/s12862-020-01713-5 (PMC7641863; doi:10.1186/s12862-020-01713-5)

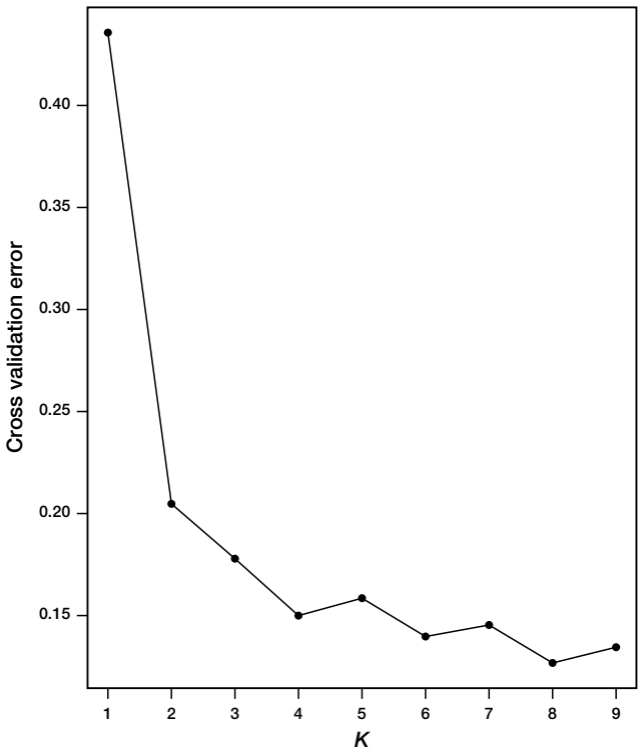

Supplement: Supplementary file 1 — Additional file 1: Fig. S1. Cross-validation errors for each K from the ADMIXTURE analyses for Japanese populations. [file 12862_2020_1713_MOESM1_ESM.pdf]

A

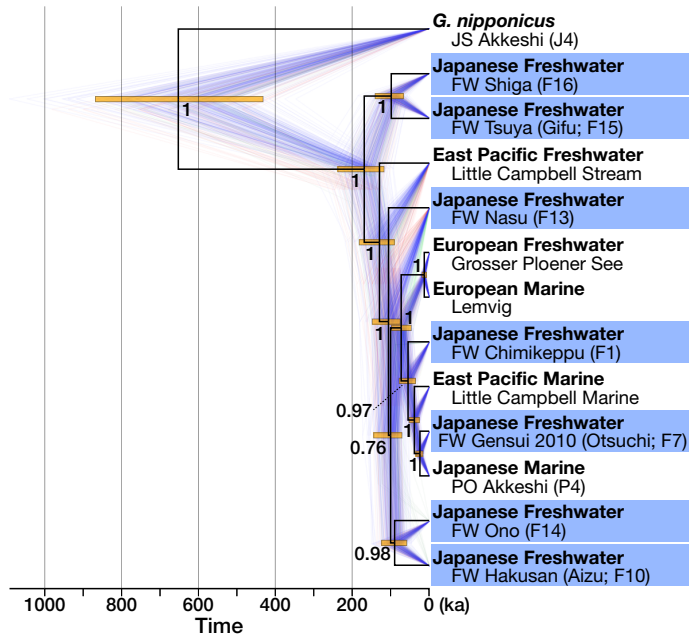

B

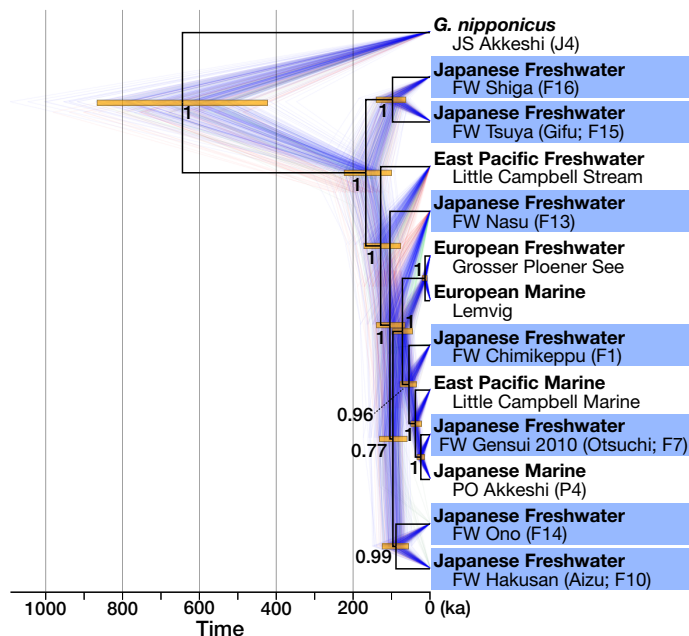

C

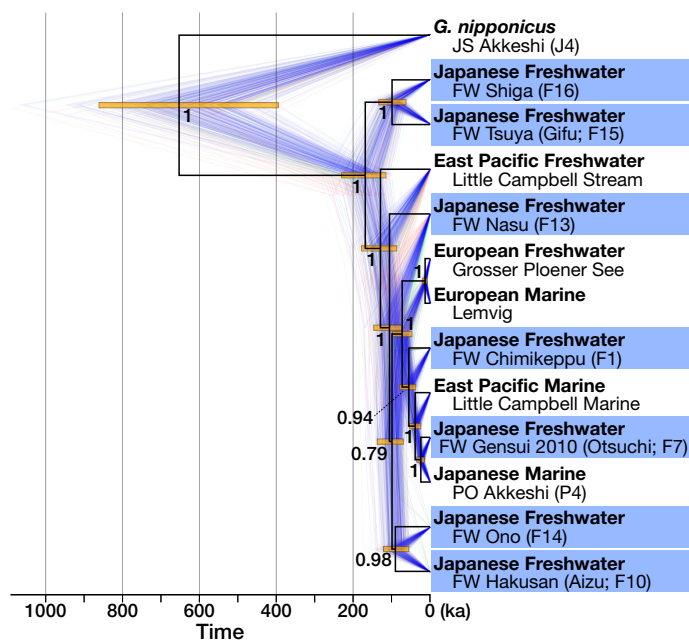

D

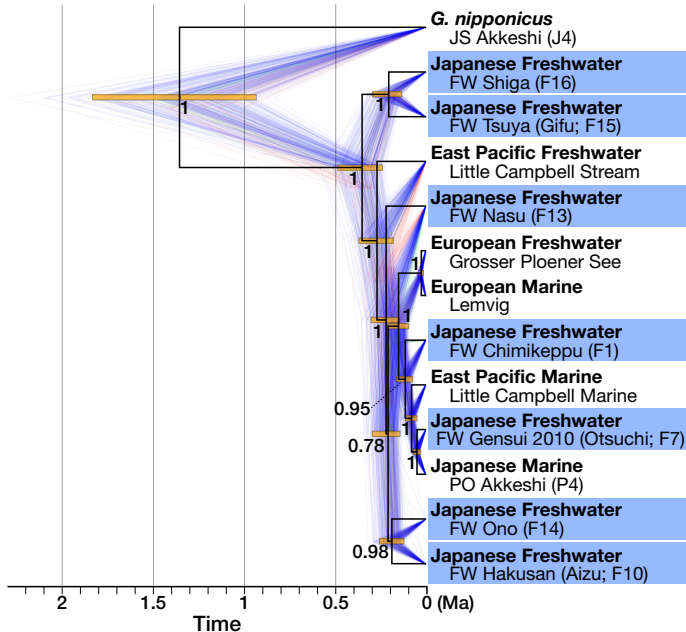

E

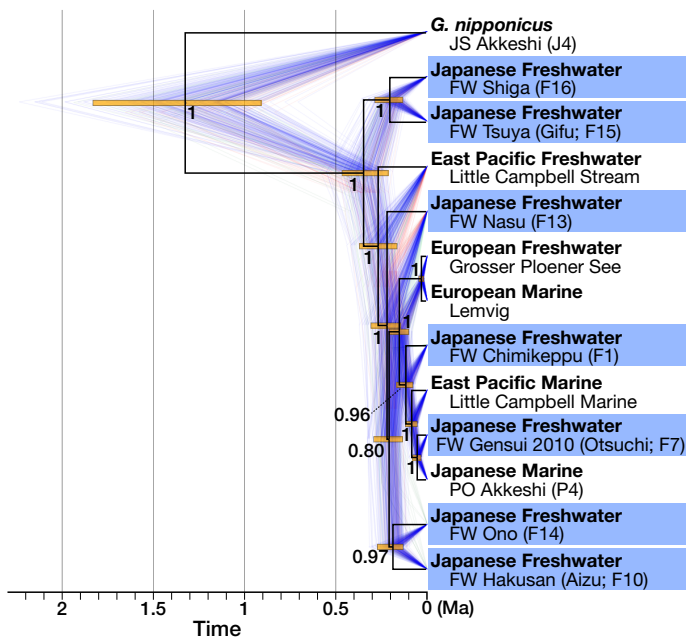

F

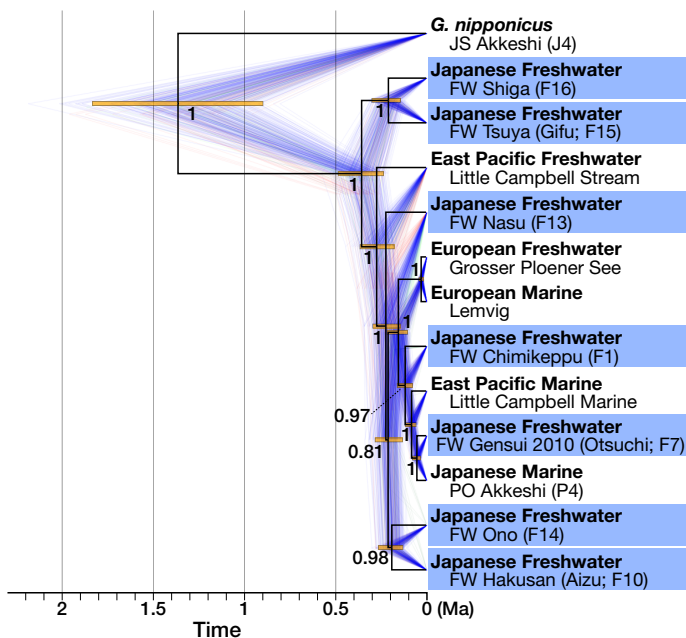

Supplement: Supplementary file 3 — Additional file 3: Fig. S3. Time-calibrated species trees of representative populations of Gasterosteus aculeatus and G. nipponicus inferred with SNAPP based on 2022 SNPs. Results of three independent runs are shown. The trees recorded in a run are overlaid by the maximum clade credibility tree. Posterior probabilities of each node are shown. Each bar indicates the 95% highest posterior density interval of the node height. (A, B, and C) Calibrated with root divergence at 680 ka BP. (D, E, and F) Calibrated with root divergence at 1.38 Ma BP. Individuals from Japanese freshwater populations of G. aculeatus are highlighted in blue. [file 12862_2020_1713_MOESM3_ESM.pdf]

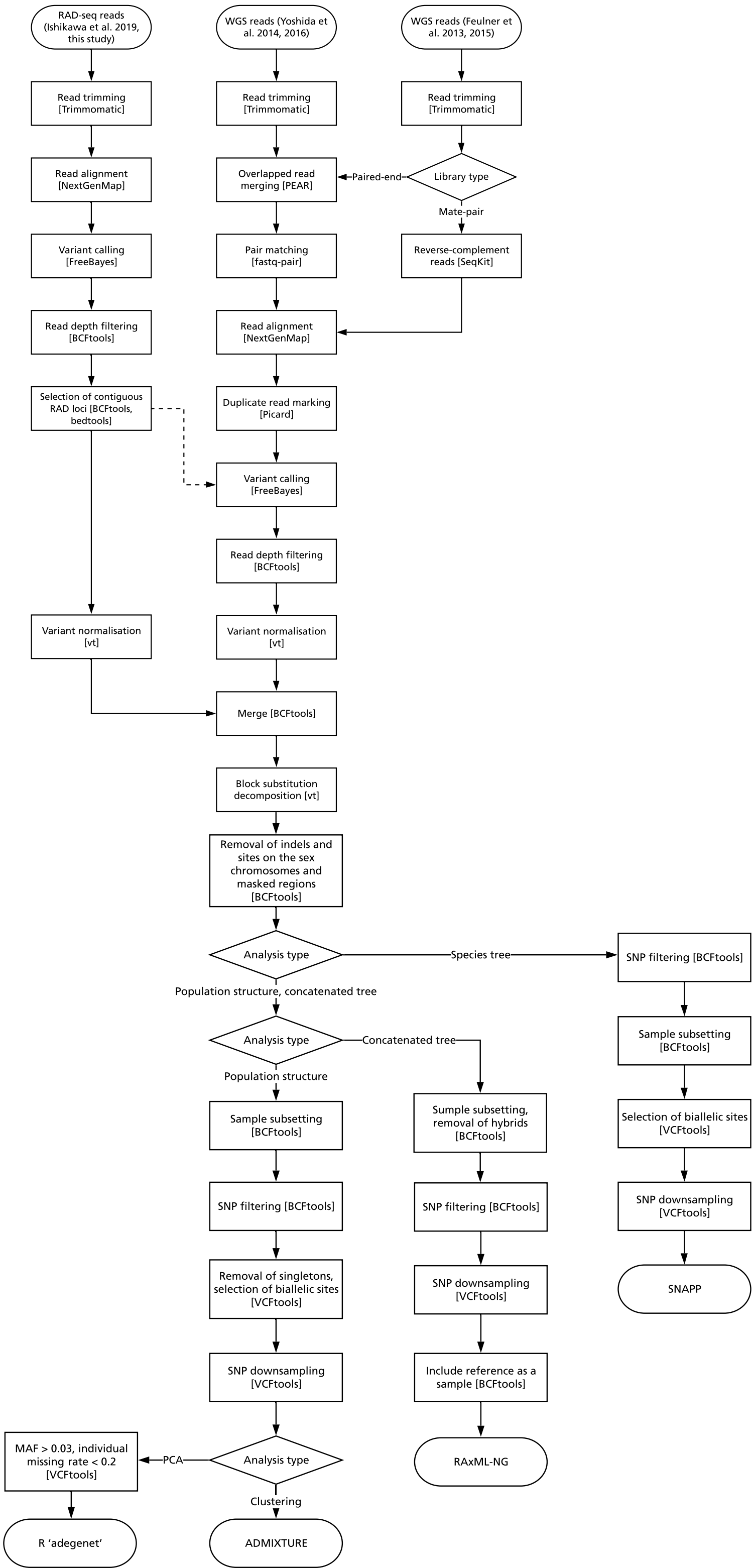

Supplement: Supplementary file 6 — Additional file 6: Fig. S4. Summary of the flow of bioinformatic analyses. [file 12862_2020_1713_MOESM6_ESM.pdf]

A

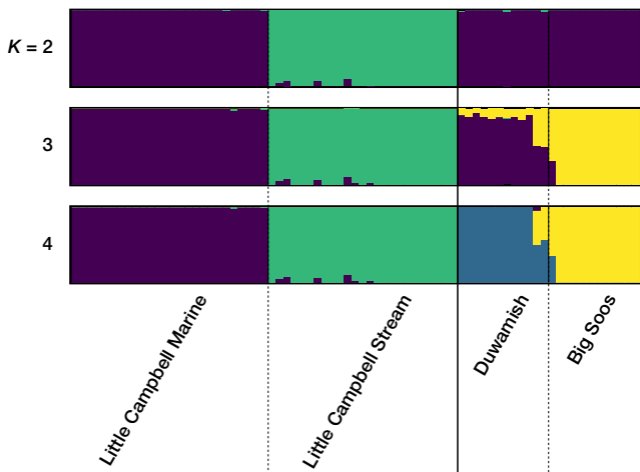

B

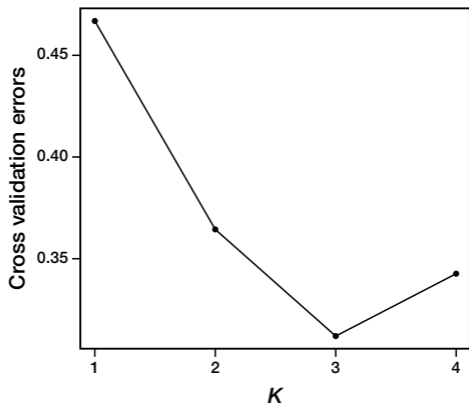

Supplement: Supplementary file 7 — Additional file 7: Fig. S5. (A) Bar plots showing the results of the population structure analyses of East Pacific samples based on 3790 SNPs with ADMIXTURE (K = 2–4). Individuals are represented as vertical bars with different colours being proportional to the genotypes belonging to each genetic cluster. (B) Cross-validation errors for each K from the ADMIXTURE analyses of the East Pacific populations. [file 12862_2020_1713_MOESM7_ESM.pdf]
