# Supplementary material for: Multiple waves of freshwater colonization of the three-spined stickleback in the Japanese Archipelago
Source: BMC Evol Biol. 2020 Nov 3;20:143. doi: 10.1186/s12862-020-01713-5 (PMC7641863; doi:10.1186/s12862-020-01713-5)
Supplement: Supplementary file 2 — Additional file 2: Fig. S2. Scatter plots of principal components of genetic differentiation in the Japanese populations based on 813 SNPs. The contributions of each principal component are shown in the parentheses. [file 12862_2020_1713_MOESM2_ESM.pdf]

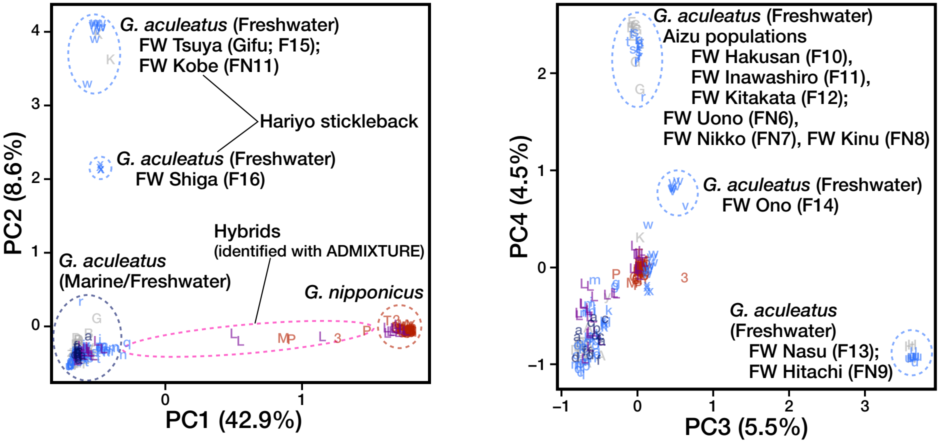

G. aculeatus (Marine)
a: PO_Choboshi (P1)
b: PO_Watarichippu (P2)
c: PO_Tokotan (P3)
d: PO_Akkeshi (P4)
e: PO_Harutori (P5)
f: PO_Fushikobetu (P6)

G. aculeatus (Freshwater)
g: FW_Chimikeppu (F1)
h: FW_Konuma (F2)
i: FW_Junsai (F3)
k: FW_Kuji (F4)
m: FW_Osanai (F5)
n: FW_Fureai (F6)
o: FW_Gensui_2010 (F7)
p: FW_Gensui_2012 (F8)
q: FW_Mast (F9)
r: FW_Hakusan (F10)
s: FW_Inawashiro (F11)
t: FW_Kitakata (F12)
u: FW_Nasu (F13)
v: FW_Ono (F14)
w: FW_Tsuya (F15)
x: FW_Shiga (F16)

G. aculeatus (Freshwater, non-native)
y: FW_Kussharo (FN1)
z: FW_Shikotsu (FN2)
A: FW_Nishitappu (FN3)
B: FW_Aisaka (FN4)
D: FW_Towada (FN5)
E: FW_Uono (FN6)
F: FW_Nikko (FN7)
G: FW_Kinu (FN8)
H: FW_Hitachi (FN9)
J: FW_Komono (FN10)
K: FW_Kobe (FN11)

G. aculeatus (Marine) / *G. nipponicus*
L: Okinebe (Oki)

G. nipponicus
M: JS_Sarufutu (J1)
N: JS_Abashiri (J2)
P: JS_Biwase (J3)
Q: JS_Akkeshi (J4)
R: JS_Harutori (J5)
S: JS_Onnechikappu (J6)
T: JS_Fushikobetu (J7)
U: JS_Ogawara (J8)
Y: JS_Kuriyama (J9)
2: JS_Benkei (J10)
3: JS_Notojima (J11)
4: JS_Mikata (J12)
5: JS_Shinji (J13)
